# Low‐energy broad‐beam photon shielding data for constituents of concrete

**DOI:** 10.1120/jacmp.v13i2.3525

**Published:** 2012-03-08

**Authors:** Folorunso O. Ogundare, Samuel A. Ogundele, Olumide O. Akerele, Fatai A. Balogun

**Affiliations:** ^1^ Department of Physics University of Ibadan Ibadan Nigeria; ^2^ National Institute of Radiation Protection and Research University of Ibadan Ibadan Nigeria

**Keywords:** attenuation coefficients, radiation shielding, soils, cement

## Abstract

The ability of concrete to attenuate ionizing radiation intensity is assessed using its linear or mass attenuation coefficient. In this work, the broad‐beam linear and mass attenuation coefficients of different types of soils and cements used for making concrete were measured at different photon energies (60–1333 keV), nearly spanning the diagnostic photon energy range, using a NaI detector. The mass attenuation coefficients of cement decreased from 0.133±0.002 at 60 keV to 0.047±0.003 at 1332.5 keV. For soils, the mass attenuation coefficient of those collected from the beach was the highest, decreasing from $0.176\pm 0.003\;{\text{cm}}^{2}/g$ at 60 keV to $0.054\pm 0.001\;{\text{cm}}^{2}/g$ at 1332.5 keV. Land soils had the least value, decreasing from $0.124\pm 0.002\;{\text{cm}}^{2}/g$ at 60 keV to $0.044\pm 0.003\;{\text{cm}}^{2}/g$ at 1332.5 keV. Limestone had smaller mass attenuation coefficients than the cement produced using it. The implication of the above is that for making concrete, beach sand should be preferred as the sand component of the concrete. Models of the form $\mu_{L}=A(E)\exp[B(E)\rho ]$ and $\mu_{m}=\alpha \ln(E)+\beta$ are proposed for fitting the linear attenuation coefficient and mass attenuation coefficient data, respectively.

PACS numbers: 87.55.N‐; 87.50.cm

## I. INTRODUCTION

Many man‐made sources of ionizing radiation abound in our technological age in addition to natural sources. These ionizing radiations have found applications in many areas including medicine, industry, research, and agriculture.^(1)^ In all these areas of applications, the use of ionizing radiation sources has been found to produce benefits but not without some detriment associated with their use. Applications in medicine have been reported as the largest contributor to population dose.^(^ ^2^ ^–^ ^4^ ^)^ In order to minimize the detriments, it has been recommended that the use of ionizing radiation should be such that radiation dose to workers, the public, and patients are as low as reasonably achievable.^(5)^

Three ways by which exposure to people are reduced include: locating the facilities away from areas where people can easily access them, minimizing the time people spend near the facilities, and shielding the facilities. The most‐used and effective way is shielding, especially in hospitals where space is limited. Shielding of rooms housing radiation facilities in hospitals or diagnostic centers is usually done using concrete or gypsum board lined with lead. Concrete is a mixture of sand, cement, and gravel. The ability of concrete to shield ionizing radiations is determined by its mass (or linear) attenuation coefficient. The higher the mass attenuation coefficients, the more efficient concrete will be in shielding ionizing radiations. The attenuation coefficient of a material depends on the attenuation coefficients of its constituents. Previous reports have assessed the shielding effectiveness by measurement of mass attenuation coefficients (MACs) of soil, cement, gravel, and limestone.^(^ ^6^ ^–^ ^8^ ^)^ These previous studies did not report the mass attenuation coefficients for some of these materials (especially cement) at any energy within diagnostic energy range. Since concrete may be used as shielding material in a radiology department, there is, therefore, the need to include energies in this range.

Experimentally, the determination of attenuation coefficients can be done using either narrow‐beam or broad‐beam geometry. One of the conditions for having narrow‐beam geometry is that the collimation of the source should be large enough just to cover the detector uniformly, thereby minimizing the number of scattered photons.^(9)^ A second condition is that both the source and attenuator should be placed at a far distance from the detector, again to minimize scattered radiations that get to the detector. In most practical applications, these conditions are not usually satisfied and the conditions applicable are broad‐beam.^(10)^ For example, in medical X‐ray imaging applications, the radiation beam is made up of primary and secondary radiations. Secondary radiation includes radiation scattered from or produced within the patient and other objects.^(^ ^11^ ^–^ ^12^ ^)^ The use of broad‐beam for the determination of attenuation coefficients of materials that are used in construction of protective barriers against radiation is therefore desirable.

The aim of this investigation is to determine the broad‐beam attenuation coefficients of soil, limestone, and cement in the energy range 60–1333 keV, which spans most of the energies used in diagnostic radiology. This study will provide attenuation coefficients data and a new model for the calculation of the attenuation coefficients of the various materials.

## II. MATERIALS AND METHODS

### A.1 Sample collection and preparation

***Soil samples***: Ordinary sand (from Akute area of Ogun State), hill soil (from Apata area of Ibadan in Oyo State), beach soil (from Eleko Beach, Victoria Island, Lagos), river soil (from Yewa River in Owode‐ketu area of Ogun State), and land soil (from Univ. of Ibadan, Oyo State). Sands from these areas are regularly collected for building construction.

***Cements*:** Cement manufactured by West Africa Portland Cement Company (WAPCO) Plc, Ewekoro, Ogun State, Nigeria), and Dangote Cement Plc (Kogi State, Nigeria) were collected.

***Limestone*:** Limestone was obtained from WAPCO, Ewekoro Cement Factory quarry plant (Ewekoro, Ogun State, Nigeria).

The samples were dried in an oven at 110°C and crushed into fine powder. The physical densities of the corresponding samples were measured by the conventional method. The crushed samples were packed into plastic containers (diameter 5.5 cm and height 6.85 cm) for gamma transmission measurement.

### A.2 Experimental procedures

**Figure 1:**
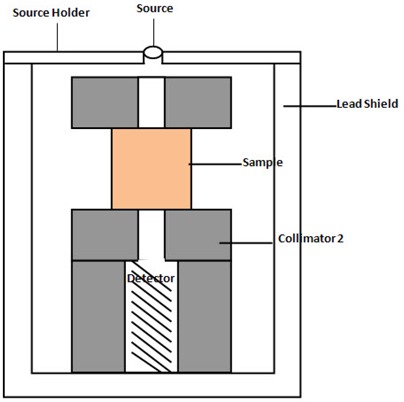
Schematic diagram for measuring the attenuation coefficients.

Gamma spectrometry using NaI detector was used to determine the attenuation of mono‐energetic gamma photons having energies 60 keV for A241m, 661.6 keV for C137s, 1173.2 and 1332.5 keV for C60o and 1274 keV for N22a by soils, cement and limestone samples. The gamma spectrometer was used at the laboratory of the National Institute of Radiation Protection and Research (University of Ibadan, Ibadan, Nigeria). The measurements were made by placing the sources (diameter 2.5 cm) at a distance of 5 cm from the sample which was 7.3 cm from the cap of the NaI detector (diameter 13 cm). A lead collimator of diameter 13 mm was placed between the source and the sample, and another one between sample and detector. The set‐up shown in Fig. 1 produced broad‐beam first because the diameter of the collimator is smaller than the diameter of the source and that of the detector, thereby causing scattered radiations reaching the detector to increase. A second reason why it is broad‐beam is that the distance between the collimator and the detector is not large enough to prevent scatter radiations from the collimator and the sample from reaching the detector. The number of counts reaching the detector with or without sample inside the plastic container was recorded for a counting time of 3600 sec.

The measured incident and transmitted intensities, together with the measured thickness of the samples, were employed in Eq. (1) to calculate attenuation coefficients:

(1) $$\mu_{L}=\frac{1}{x}ln(I_{O}/I)$$

where μ is the linear attenuation coefficient in ${\text{cm}}^{-1}$ and *x* is the thickness of the sample in cm. $I_{o}$ is the gamma ray counts with the empty plastic container, and *I* is the gamma ray count with sample filled plastic container. The mass attenuation coefficients (μ/ρ) in ${\text{cm}}^{2}/g$ for the collected materials were obtained from Eq. (2) by dividing the average linear attenuation coefficient with the measured density (ρ) of the sample:

(2) $$\mu_{m}=\frac{\mu }{\rho }=\frac{1}{\rho x}ln(I_{O}/I)$$

## III. RESULTS & DISCUSSION

**Figure 2:**
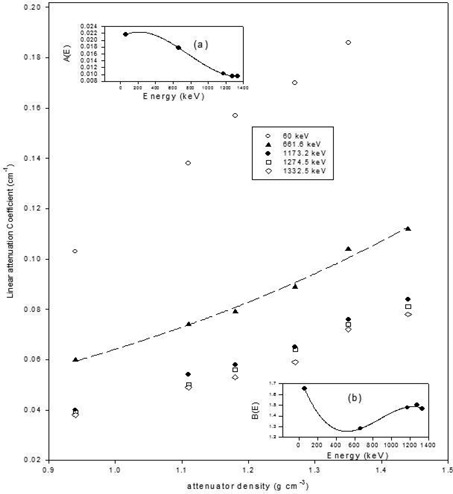
A plot of linear attenuation coefficient against density (dash lines represent a typical exponential fit to the plot). Cubic polynomial fit to the plots of fitting constants A(E) versus energy and B(E) versus energy are shown in the insets (a) and (b), respectively.

The average of the measured linear attenuation coefficients and densities for each of the samples are indicated in Table 1. The highest percentage standard deviation in the measured linear attenuation coefficient values is 6.7%. The relationship between the linear attenuation coefficient $\mu_{L}$, energy *E* and density ρ was best described by the following equation:

(3) $$\mu_{L}=A(E)\exp[B(E)\rho ]$$

where $A(E)=({\text{aE}}^{3}+{\text{bE}}^{2}+\text{cE}+d)$ and $B(E)=({\text{eE}}^{3}+{\text{fE}}^{2}+\text{gE}+h)$. The fitting coefficients a, b, c, d, e, f, g, and h are given in Table 2. As shown in Fig. 2, Eq. (3) provides a good fit to the data.

**Table 1:** Measured values of linear attenuation coefficients $\left({\text{cm}}^{-1}\right)$ for different concrete constituents.

*Energy (keV)*	*Limestone* $\rho =0.94g/{\text{cm}}^{3}$	*Beach Soil* $1.44g/{\text{cm}}^{3}$	*Ordinary Sand* $1.35g/{\text{cm}}^{3}$	*River Soil* $1.18g/{\text{cm}}^{3}$	*Hill Soil* $1.35g/{\text{cm}}^{3}$	*Land Soil* $1.11g/{\text{cm}}^{3}$	*Cement* $1.27g/{\text{cm}}^{3}$
60	0.103±0.001	0.253±0.002	0.201±0.003	0.157±0.003	0.186±0.002	0.138±0.001	0.170±0.002
661.6	0.060±0.002	0.112±0.001	0.104±0.002	0.079±0.005	0.102±0.002	0.074±0.005	0.089±0.003
1173.2	0.040±0.002	0.084±0.002	0.076±0.001	0.058±0.0003	0.076±0.0004	0.054±0.001	0.065±0.001
1274.5	0.039±0.002	0.081±0.003	0.074±0.002	0.056±0.002	0.073±0.0004	0.050±0.001	0.064±0.003
1332.5	0.038±0.002	0.078±0.001	0.072±0.002	0.053±0.007	0.070±0.0004	0.049±0.0004	0.059±0.003

**Table 2:** Values of the fitting parameters in the function $\mu_{L}=A(E)\text{exp}[B(E)\rho ]$.

*Parameter*	*Value*
a	$1.825\times 10^{-11}$
b	$-4.216\times 10^{-8}$
c	$1.517\times 10^{-5}$
d	$2.094\times 10^{-2}$
e	$1.252\times 10^{-9}$
f	$3.282\times 10^{-6}$
g	$-2.385\times 10^{-3}$
h	1.785

The mass attenuation coefficients of the soil, limestone, and cement samples across the energy range 60–1332.5keV are presented in Table 3. Among the soil samples, beach soil had the highest mass attenuation coefficient value, decreasing from $0.176\pm 0.003\;g/{\text{cm}}^{3}$ at 60 keV to $0.054\pm 0.001\;{\text{cm}}^{3}/g\;1332.5\;\text{keV}$, while land soils had the least value of all the energies, decreasing from $0.124\pm 0.002\;g/{\text{cm}}^{3}$ at 60 keV to $0.044\pm 0.0003\;{\text{cm}}^{3}/g$ at 1332.5 keV. Limestone had smaller mass attenuation coefficient than the cement produce from it. The higher value for the cement compared to the limestone indicated that the processing of the limestone into cement improved its shielding ability. The equation that best described the variation of mass attenuation coefficient with energy was found to be

(4) $$\mu_{m}=\alpha \ln(E)+\beta$$

where the fitting coefficients α and β are as listed in Table 4. Unlike this logarithm equation, Awadallah and Imran^(8)^ reported a power relation between mass attenuation coefficient and energy. In formulating the present equation, an energy (60 keV) lower than the limit of 100 keV reported for the validity of the power relation of Awadallah and Imran was included. The present equation may therefore be more accurate than their power relation. Furthermore, unlike the Awadallah and Imran equation that is not valid in the diagnostic energy range, the present model is valid.

**Table 3:** Measured values of mass attenuation coefficients $\left({\text{cm}}^{2}/g\right)$ for different concrete constituents.

*Energy (keV)*	*Limestone* $\rho =0.94g/{\text{cm}}^{3}$	*Beach Soil* $1.44g/{\text{cm}}^{3}$	*Ordinary Sand* $1.35g/{\text{cm}}^{3}$	*River Soil* $1.18g/{\text{cm}}^{3}$	*Hill Soil* $1.35g/{\text{cm}}^{3}$	*Land Soil* $1.11g/{\text{cm}}^{3}$	*Cement* $1.27g/{\text{cm}}^{3}$
60	0.109±0.002	0.176±0.003	0.149±0.001	0.133±0.000	0.138±0.003	0.124±0.002	0.133±0.002
661.6	0.064±0.001	0.078±0.002	0.077±0.002	0.067±0.004	0.076±0.001	0.067±0.002	0.070±0.002
1173.2	0.043±0.002	0.058±0.001	0.056±0.001	0.049±0.000	0.056±0.0003	0.049±0.001	0.051±0.001
1274.5	0.041±0.002	0.056±0.002	0.055±0.001	0.047±0.004	0.054±0.003	0.045±0.001	0.050±0.003
1332.5	0.040±0.002	0.054±0.001	0.053±0.000	0.045±0.004	0.052±0.0003	0.044±0.0003	0.047±0.003

**Table 4:** Values of the fitting parameters in the function $\mu_{m}=\alpha \ln(E)+\beta$ for the investigated samples.

*Material*	α	β
Limestone	−0.0221	0.2010
Beach Soil	−0.0395	0.3370
Ordinary Sand	−0.0310	0.2761
River Soil	−0.0282	0.2489
Hill Soil	−0.0275	0.2515
Land Soil	−0.0256	0.2296
Cement	−0.0276	0.2463

The average of the measured mass attenuation coefficient values of similar samples at the same energy in different countries are presented in Table 5. For limestone, the MAC value from Bangladesh^(7)^ agreed very well with the present value at all energies, while those reported from Jordan^(8)^ were significantly higher, with percentage difference that ranged between 25.58% to 36.76%. The samples from Jordan might, therefore, have contained some other materials that the authors were not able to separate. The measured attenuation coefficient in Nigeria, Bangladesh, and Brazil for cement and soil samples indicated high similarities with the highest percentage difference of about 8%. The general agreement among the values from the different countries implied that Eqs. (3) and (4) can be used for soils from any location.

**Table 5:** Comparison of mass attenuation coefficients for sampled soil and sand in different countries.

	*Attenuation Coefficient for:*				
*Material*	*661.6 keV*	*1173.2 keV*	*1332.5 keV*	*Country*	*References*
Limestone	0.060±0.002	0.042±0.002	0.040±0.002	Bangladesh	Alam et al.^(7)^
	0.086±0.002	0.068±0.005	0.055±0.001	Jordan	Awadalah and Imran^(8)^
	0.064±0.001	0.043±0.002	0.040±0.000	Nigeria	Present work
Beach Soil	0.076±0.002	0.056±0.002	0.052±0.002	Bangladesh	Alam et al.^(7)^
	0.080±0.002	‐	0.058±0.001	Brazil	Appoloni and Rios^(15)^
	0.078±0.009	0.058±0.001	0.054±0.001	Nigeria	Present work
Ordinary Sand	0.077±0.002	0.058±0.002	0.054±0.002	Bangladesh	Alam et al.^(7)^
	0.077±0.002	0.056±0.001	0.053±0.0001	Nigeria	Present work
River Soil	0.070±0.002	0.046±0.002	0.049±0.002	Bangladesh	Alam et al.^(7)^
	0.067±0.004	0.049±0.0002	0.045±0.004	Nigeria	Present work
Hill Soil	0.075±0.002	0.055±0.001	0.050±0.001	Bangladesh	Alam et al.^(7)^
	0.076±0.001	0.056±0.003	0.052±0.003	Nigeria	Present work
Land Soil	0.072±0.002	0.053±0.001	0.048±0.001	Bangladesh	Alam et al.^(7)^
	0.069±0.005	0.049±0.001	0.044±0.003	Nigeria	Present work
Cement	0.071±0.002	0.050±0.002	0.047±0.002	Bangladesh	Alam et al.^(7)^
	0.070±0.002	0.051±0.003	0.047±0.003	Nigeria	Present work

For applicability of the results from this study for shielding calculations in applications with polyenergetic radiation sources, effective energy which is essentially an estimate of the penetration power of the X‐ray beam is used.^(10)^ Effective energy is the energy of a monoenergetic source that will give the same half value layer as the polyenergetic X‐ray beam.^(^ ^13^ ^–^^115^

## IV. CONCLUSIONS

Broad‐beam linear and mass attenuation coefficients of soils and cements from Southwest Nigeria have been determined at energies including those in the diagnostic energy range. Beach soil was found to be the best sand type for making concrete for the purpose of ionizing radiation shielding. Limestone has smaller mass attenuation coefficients than the cement produced using it. Models for calculating linear and mass attenuation coefficient values of soils and cements given the density of the sample and the energy of the radiation source were developed.
